# EUS-guided transmural treatment of afferent loop syndrome: a systematic review and meta-analysis

**DOI:** 10.3389/fgstr.2026.1853386

**Published:** 2026-07-14

**Authors:** Thomas Balanis, Michael Doulberis, Radu Tutuian

**Affiliations:** 1 Clinic for Gastroenterology and Hepatology, Bürgerspital Solothurn, Solothurn, Switzerland; 2 Faculty of Medicine, University of Zurich, Zurich, Switzerland; 3 Department of Gastroenterology and Hepatology, University Hospital Zurich, Zurich, Switzerland; 4 Division of Gastroenterology and Hepatology, Kantonsspital Aarau, Aarau, Switzerland; 5 Faculty of Medicine, University of Bern, Bern, Switzerland; 6 Carol Davila University of Medicine and Pharmacy, Bucharest, Romania

**Keywords:** afferent loop syndrome, endoscopic ultrasound, enteroenterostomy, EUS-guided gastroenterostomy, lumen-apposing metal stent

## Abstract

**Background:**

Afferent loop syndrome (ALS) is an uncommon but clinically relevant complication after pancreaticobiliary or upper gastrointestinal reconstruction, most often in patients with recurrent malignant disease. Endoscopic ultrasound-guided creation of a transmural bypass, either as gastroenterostomy or enteroenterostomy, has emerged as a minimally invasive alternative to surgery or percutaneous drainage. We aimed to systematically review the available evidence and provide a pooled descriptive analysis of the efficacy and safety of EUS-guided treatment for ALS.

**Methods:**

A systematic review was performed in accordance with PRISMA principles. PubMed/MEDLINE, Embase and the Cochrane Library were searched up to 15 May 2025 for studies reporting EUS-guided gastroenterostomy or enteroenterostomy for ALS. Case reports, case series and observational studies with extractable outcome data were eligible. Data on study design, indication, stent type, technical success, clinical success, adverse events and follow-up were extracted and synthesized.

**Results:**

Twelve studies involving 134 patients were included. On crude analysis, technical success was achieved in 132/134 patients (98.5%), clinical success in 127/134 (94.8%), and adverse events were reported in 13/134 (9.7%). In the pooled random-effects analysis, the technical success rate was 93.5% (95% CI 87.1–96.8; I²=0%), the clinical success rate was 91.0% (95% CI 84.5–94.9; I²=0%), and the overall adverse-event rate was 14.6% (95% CI 9.1–22.5; I²=0%). Adverse events were mainly procedure-related pain, fever, stent misdeployment, peritonitis or intra-abdominal infection. Electrocautery-enhanced lumen-apposing metal stents were used in most studies, whereas fully covered self-expandable metal stents were used in a smaller subset. Reported follow-up ranged from 1 to 15 months.

**Conclusions:**

EUS-guided transmural bypass represents a promising, minimally invasive, and technically feasible alternative for the management of ALS. However, given the retrospective nature and small sample sizes of the available evidence, larger comparative trials are warranted to define its definitive role.

## Introduction

Afferent loop syndrome (ALS) is a rare but potentially severe complication after upper gastrointestinal and pancreaticobiliary reconstructive surgery, particularly after pancreaticoduodenectomy or Roux-en-Y reconstruction (1–3). Obstruction of the afferent limb leads to accumulation of biliary, pancreatic and enteric secretions, which may result in abdominal pain, nausea, vomiting, jaundice, cholangitis or pancreatitis (4, 5). In oncologic patients, ALS is often related to recurrent or progressive malignancy and may occur in a palliative setting where conventional surgery is undesirable or no longer feasible (1, 6).

Historically, management options have included surgical revision, percutaneous drainage and enteroscopy-assisted luminal stenting (6–8). However, these approaches may be technically difficult, invasive or associated with substantial morbidity in patients with altered anatomy, advanced cancer and limited physiological reserve (1, 6, 8). In recent years, therapeutic endoscopic ultrasound (EUS) has expanded the spectrum of minimally invasive internal drainage procedures in such patients (6, 9).

EUS-guided gastroenterostomy (EUS-GE) or enteroenterostomy (EUS-EE) allows creation of a bypass between the obstructed afferent limb and the stomach or adjacent small bowel (10–13). In most contemporary reports, this has been performed using electrocautery-enhanced lumen-apposing metal stents (EC-LAMS), although fully covered self-expandable metal stents (FCSEMS/SEMS) have also been used in some series (14–18). The theoretical advantages of EUS-guided internal bypass include rapid decompression, avoidance of external drains, and a less invasive alternative to repeat surgery (6, 9, 13).

However, evidence remains fragmented across small case series and observational studies, and no dedicated pooled analysis of efficacy and safety exists (10–18). The aim of the present study was therefore to systematically review the available literature on EUS-guided transmural bypass procedures (including gastroenterostomy and enteroenterostomy) of ALS and to summarize technical success, clinical success, adverse events and procedural characteristics across the included studies.

## Methods

### Study design and protocol

This study was designed as a systematic review with pooled study-level analysis and was conducted in accordance with the PRISMA 2020 statement (19). A pre-specified protocol was submitted to the International Prospective Register of Systematic Reviews (PROSPERO, CRD420261345580) (20). The review question was structured according to the PICO framework (21).

### Eligibility criteria

Studies were eligible if they reported adult patients (≥18 years) with ALS treated by EUS-guided transmural creation of a gastrointestinal bypass, including EUS-guided gastroenterostomy (EUS-GE) or enteroenterostomy/jejunojenunostomy (EUS-EE/EUS-JJ). Case reports, case series and observational cohort studies were considered eligible if extractable data on procedural or clinical outcomes were available.

Studies were excluded if they focused exclusively on surgical, percutaneous or non-EUS endoscopic management without separate outcome data for EUS-guided treatment. Conference abstracts without sufficient extractable data were also excluded from the final pooled analysis.

### Literature search

A comprehensive electronic literature search was performed in PubMed/MEDLINE, Embase and the Cochrane Library through May 2025. The search strategy combined terms related to “afferent loop syndrome”, “afferent limb syndrome”, “endoscopic ultrasound”, “EUS-guided gastroenterostomy”, “jejunojejunostomy”, “gastrojejunostomy”, and “lumen-apposing metal stent”. The search strategy was adapted for each database. The full Boolean search string for each database is enclosed in supplementary **Supplementary Table 1**. Additional manual screening of the reference lists of relevant articles was also performed.

### Study selection

**Figure 1:**
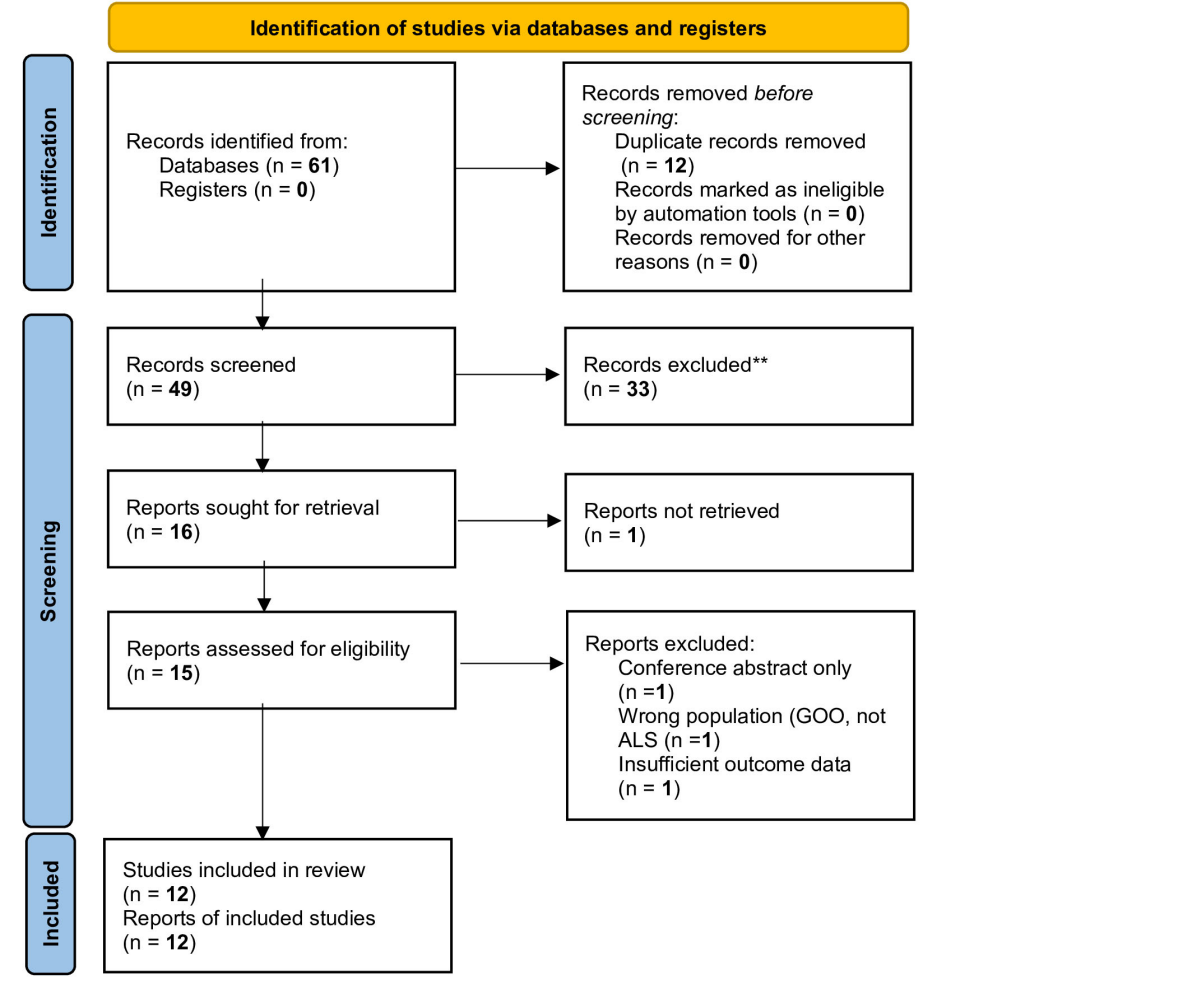
PRISMA 2020 flow diagram for new systematic reviews which included searches of databases and registers only. Excluded studies with argumentation is provided. ALS, afferent loop syndrome; EUS, endoscopic ultrasound; LAMS, lumen-apposing metal stent; PRISMA, Preferred Reporting Items for Systematic Reviews and Meta-Analyses.

After removal of duplicates, two reviewers (TB, MD) independently screened titles and abstracts for relevance. Full-text review was subsequently performed for potentially eligible studies. Discrepancies were resolved by discussion and, when necessary, consultation with a third senior reviewer (RT). The study selection process is summarized in the PRISMA flow diagram (**Figure 1**).

### Risk of bias assessment

Risk of bias was assessed independently by two reviewers (TB, MD) according to study design. Case reports and small case series were evaluated using the National Institutes of Health quality assessment tools (22), whereas retrospective and prospective non-randomized studies were assessed using ROBINS-I (**Supplementary Figure 1**) (23).

### Data extraction

Data extraction was performed independently by two reviewers (TB, MD), with discrepancies resolved by consensus through the senior author (RT). The following variables were extracted from each eligible study: first author, year of publication, study design, number of patients, underlying etiology, type of EUS-guided procedure, stent type, technical success, clinical success, adverse events, and follow-up. When available, additional procedural characteristics, including access route, stent diameter, and placement of coaxial double-pigtail plastic stents, were also recorded.

Technical success was defined as successful transmural creation and deployment of the EUS-guided anastomosis. Clinical success was defined as the complete resolution or significant improvement of ALS-related symptoms and/or radiologic decompression of the afferent limb, typically within 24-72h post-interventionally, according to the definitions used in the individual studies.

### Data synthesis and statistical analysis

Crude event rates were calculated by dividing the total number of events by the total number of treated patients across the included studies. In addition, exploratory pooled event rates with 95% confidence intervals were estimated using a random-effects model according to DerSimonian and Laird (24). Statistical heterogeneity was assessed using the I² statistic (25, 26). To account for studies with 0% or 100% event rates, a logit transformation was applied with a standard continuity correction of 0.5 added to the cells with zero events. Owing to the predominance of case reports and small retrospective series, the pooled analysis was considered exploratory. All analyses were conducted using R software (version 4.3.0) with the ‘meta’ package, prioritizing pooled estimates over crude rates for final interpretation. Publication bias was not formally assessed due to the small number of included studies.

### Quality of evidence

The overall certainty of evidence was evaluated according to GRADE principles (27). Given the observational design of the included studies, the certainty of evidence was expected to be low to very low.

## Results

### Study selection and study characteristics

A total of 61 records were identified through database searching. After removal of duplicates and screening, 49 full-text articles were assessed for eligibility. Of these, 12 studies were included in the final analysis, while conference abstracts without sufficient extractable outcome data and studies with inappropriate populations were excluded (31). The study selection process is shown in **Figure 1**.

The final dataset comprised 12 studies and 134 patients treated with EUS-guided transmural drainage for ALS. Included publications were case reports or retrospective case series, with a smaller number of multicenter observational studies. The majority of patients had malignant ALS, whereas a minority had benign or mixed etiologies. A detailed summary of the included studies is provided in **Table 1**.

**Table 1:** Summary of included studies.

Study	Year	Country	Study design	Patients (n)	Indication	Stent type	Stent (mm) diameter	Technical success*	Clinical success*	AE	Follow-up (months)
Benallal et al.	2018	Europe	Case report	1	Malignant	EC-LAMS	15	100% (1/1)	100% (1/1)	None	3
Lee et al.	2022	South Korea	Case series	2	Benign / Malignant	EC-LAMS	20	100% (2/2)	100% (2/2)	None	2–5
De Bie et al.	2021	Europe	Case series	6	Malignant	EC-LAMS	20	100% (6/6)	100% (6/6)	None	2–12
Taunk et al.	2015	USA	Case series	3	Malignant	EC-LAMS	15	100% (3/3)	100% (3/3)	None	1
Rodrigues-Pinto et al.	2016	USA	Case series	4	Malignant	EC-LAMS	20	100% (4/4)	100% (4/4)	None	2–6
Matsubara et al	2023	Japan	Case series	12	Malignant	Fc-SEMS	10	100% (12/12)	100% (12/12)	1	3
Hagiwara, et al.	2024	Japan	Case series	25	Malignant	SEMS	10	100% (25/25)	96% (24/25)	6 (3 abdominal pain, Abscess, peritonitis)	15
Yamamoto et al.	2024	Japan	Case series	3	Malignant	EC-LAMS	15	100% (3/3)	100% (3/3)	1 fever	2–3
Rizzo et al.	2025	Europe	Case series	14	Malignant	EC-LAMS	20	100% (14/14)	100% (14/14)	None	5
Ligresti et al.	2020	Italy	Case report	1	Malignant	EC-LAMS	20	100% (1/1)	100% (1/1)	None	3
Brewer-Gutierrez et al.	2018	USA	Multicentre case series	18	Benign/ Malignant	EC-LAMS	20	100% (18/18)	88.9% (16/18)	3 AE (abdominal pain)	3–6
Pérez-Cuadrado-Robles et al.	2022	Europe	Multicentre observational study	45	Malignant	EC-LAMS	≥15 vs <15	95.6% (43/45)	91.1% (41/45)	2 AE(LAMS misdeployment)	Median 4 months

AE, adverse event, EC-LAMS, electrocautery-enhanced lumen-apposing metal stent.

*Note on Outcome Definitions: Technical success was uniformly defined across studies as the successful transmural deployment of the stent (LAMS or SEMS) establishing a patent anastomosis. Clinical success was defined as the complete or significant resolution of ALS symptoms (nausea, vomiting, abdominal pain) and/or lab/radiologic improvement (e.g., relief of loop distension or decreased bilirubin) typically captured within 24 to 72 hours post-procedure.

### Procedural characteristics

EUS-guided treatment was performed either as gastroenterostomy or as enteroenterostomy, depending on postoperative anatomy and the relationship of the dilated afferent limb to the gastric or enteral lumen. Most contemporary studies used electrocautery-enhanced lumen-apposing metal stents, whereas fully covered self-expandable metal stents were used in selected series. Reported stent diameters ranged from 10 to 20 mm. Fluoroscopic guidance and coaxial double-pigtail plastic stent placement were described inconsistently and could not be analyzed systematically.

### Technical success

**Figure 2:**
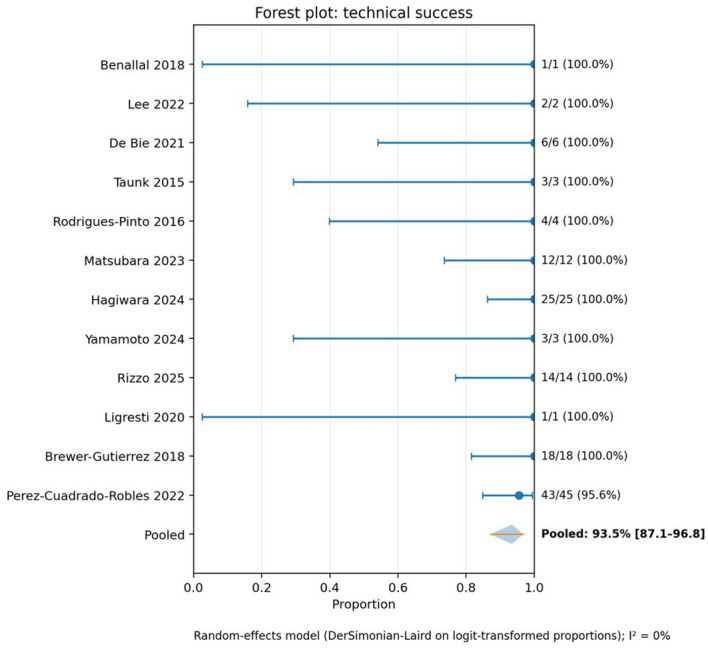
Forrest plot of technical success.

Technical success was reported in all included studies and was achieved in 132 of 134 patients (98.5%). In the pooled random-effects analysis, the technical success rate was 93.5% (95% CI 87.1–96.8; I²=0%). Most studies reported complete technical success, with failures limited to a small number of cases in the larger multicenter cohorts. Overall, these findings indicate a high degree of procedural feasibility across different centers and stent platforms. The forest plot for technical success is displayed in **Figure 2**.

### Clinical success

**Figure 3:**
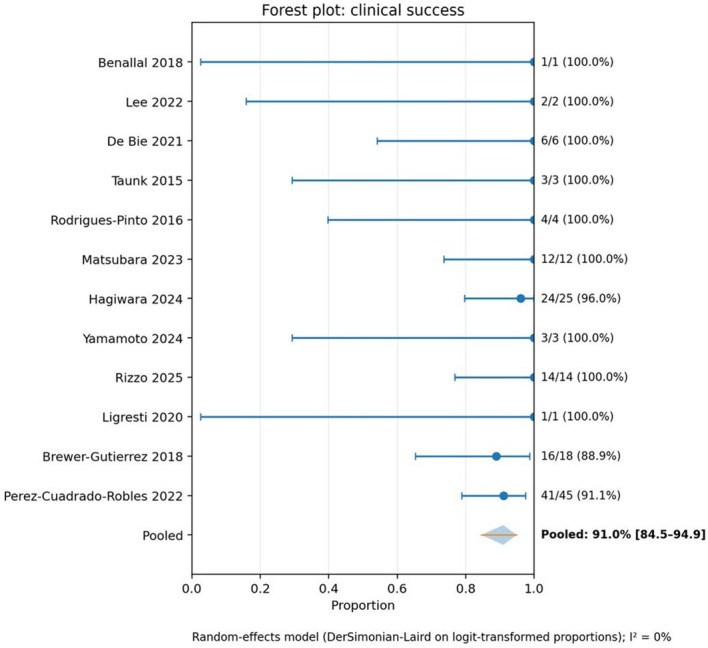
Forrest plot of clinical success.

Clinical success was achieved in 127 of 134 patients (94.8%). In the pooled random-effects analysis, the clinical success rate was 91.0% (95% CI 84.5–94.9; I²=0%). In most studies, clinical improvement occurred rapidly after successful creation of the anastomosis and was reflected by resolution of obstructive symptoms and decompression of the afferent limb. Although definitions of clinical success were not entirely uniform across studies, the overall pattern was highly consistent and favored EUS-guided internal bypass. The forest plot for clinical success is shown in **Figure 3**.

### Adverse events

**Figure 4:**
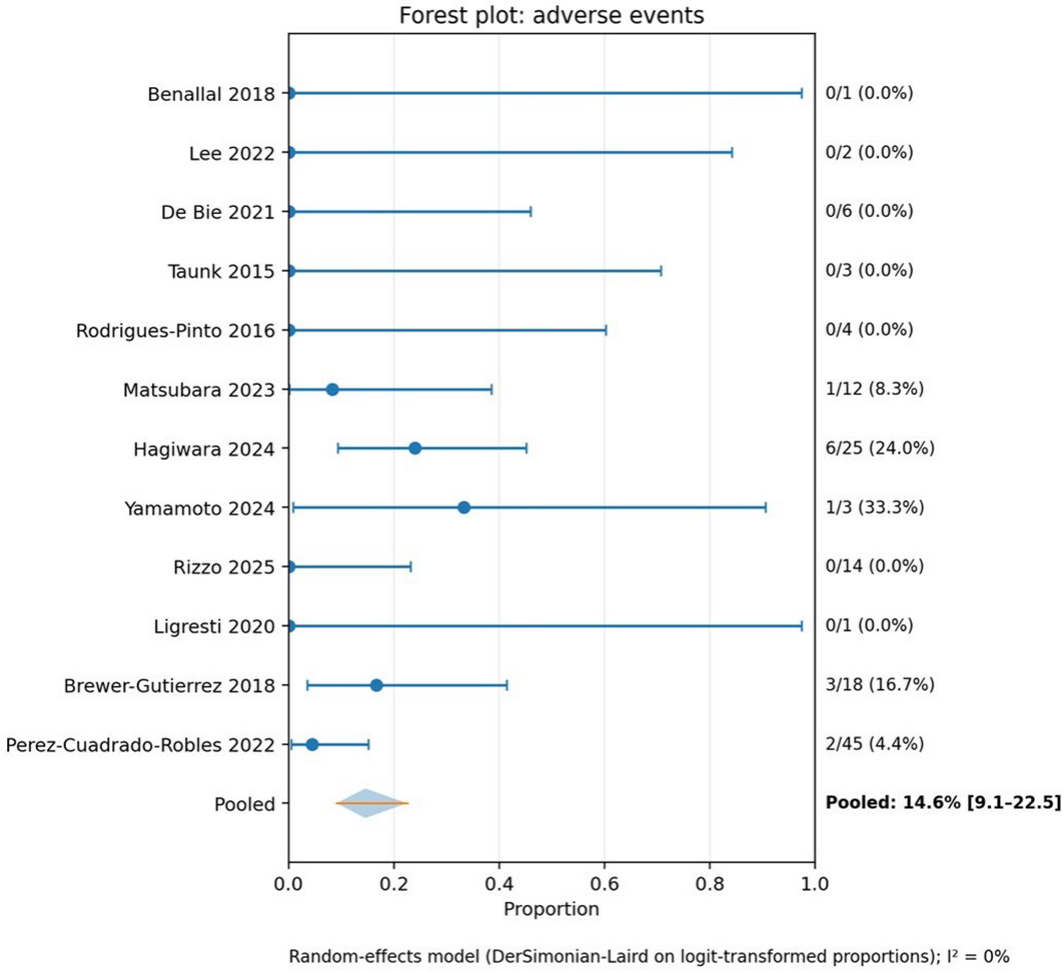
Forrest plot of adverse events.

Adverse events were reported in 13 of 134 patients (crude rate, 9.7%). In the pooled random-effects analysis, the overall adverse-event rate was 14.6% (95% CI 9.1–22.5; I²=0%). The forest plot of this analysis is enclosed in **Figure 4**. Reported events included abdominal pain, fever, stent misdeployment, peritonitis, intra-abdominal infection and abscess formation. Most studies reported either no adverse events or only a small number of complications, although higher adverse-event rates were described in some larger contemporary series. Due to the fact that adverse-event definitions and grading were not fully standardized across studies, the results should be interpreted with caution.

### Follow-up

Follow-up duration varied substantially across the included studies and was inconsistently reported, ranging approximately from 1 to 15 months. In patients with malignant ALS, EUS-guided treatment was generally performed with palliative intent and was associated with short-term symptomatic relief. Longer-term outcomes in benign ALS could not be reliably assessed because of the limited number of such cases and heterogeneity in follow-up reporting.

### Reintervention and recurrence

Reintervention and recurrence were not uniformly reported across the included studies and was subsequently not feasible to pool them as formal secondary endpoints in the present analysis. Once described, reinterventions were mainly related to stent dysfunction, migration or recurrent obstructive symptoms. Of note, key long-term parameters—such as the exact duration of stent patency and the durability of symptomatic relief—were majorly absent or qualitatively described in a non-standardized manner across the available literature, preventing a robust quantitative synthesis of these relevant longitudinal outcomes.

### Risk of bias and quality of evidence

Risk of bias was assessed using the ROBINS-I tool for the 10 retrospective cohort and case−series studies, and the NHLBI Quality Assessment Tool for the two case reports (Benallal et al., Ligresti et al.). The detailed ROBINS-I judgements for each of the 10 studies are presented in **Supplementary Figure 1**. Overall, all 10 non−randomized studies were judged to have moderate to serious risk of bias due to confounding. The two case reports were rated as high risk of bias across all domains because of their single−patient, non−comparative, descriptive design.

Additionally, a formal evaluation of the certainty of evidence was performed using the GRADE methodology for the primary clinical endpoints (technical success, clinical success, and adverse events). Reflecting the cumulative impact of the retrospective designs, small sample sizes, and highly probable publication bias, the overall certainty of evidence for all major outcomes was uniformly graded as ‘Very Low’ (**Supplementary Table 2**).

## Discussion

The findings of this systematic review and meta-analysis support that EUS-guided transmural bypass for ALS achieves excellent initial technical and clinical success with a favorable safety profile. Nevertheless, these remarkably high success rates must be interpreted with cumulative caution, since the current evidence base consists entirely of retrospective case reports and small series, which are inherently prone to both selection and publication bias. Consequently, the high success rates observed may partially reflect the limitations of small-study meta-analyses rather than absolute clinical homogeneity. The abovementioned findings suggest that EUS-guided gastroenterostomy or enteroenterostomy presents a promising and feasible minimally invasive treatment option in a particularly complex patient population, often characterized by altered postoperative anatomy, advanced malignancy and limited suitability for repeat surgery (10–18, 30, 31).

An important finding of the present review is the consistency of efficacy across heterogeneous study designs. Despite the predominance of case reports, small retrospective series and multicenter observational cohorts, most studies reported near-complete technical and clinical success. This suggests that, when the dilated afferent limb can be adequately identified and accessed endosonographically, creation of a transmural bypass provides reliable decompression and rapid symptom relief. From a practical clinical perspective, this is highly relevant, as patients with ALS frequently present with abdominal pain, nausea, vomiting, obstructive jaundice or cholangitis and often require prompt palliation rather than escalation to more invasive rescue strategies (1, 4–6).

The current dataset also reflects the technical evolution of EUS-guided management of ALS. Most patients were treated using electrocautery-enhanced lumen-apposing metal stents, which have become the preferred platform for single-step transmural anastomosis creation (10, 13, 14, 17, 18). However, two more recent series used fully covered self-expandable metal stents and also reported favorable outcomes (15, 16). This broadens the clinical relevance of the present review, as it indicates that successful EUS-guided bypass is not exclusively dependent on a single stent platform, but rather on the combination of appropriate patient selection, favorable anatomy and operator expertise. At the same time, EC-LAMS remain particularly attractive because they simplify the procedure and may reduce the need for accessory exchanges (6, 9).

From a clinical standpoint, the majority of cases in the available literature were malignant ALS, most commonly occurring after surgery for pancreaticobiliary malignancies (1, 6, 16, 28). In this setting, EUS-guided bypass appears especially valuable because it offers internal drainage without the burden of external catheters and avoids the morbidity of repeat surgery in patients with advanced disease and limited physiological reserve (6, 8, 29). In contrast, benign ALS remains underrepresented in the literature. Although the available reports suggest that EUS-guided treatment may also be effective in selected benign cases, the current evidence is insufficient to define long-term durability, optimal stent dwell time and the role of elective stent removal in this subgroup (6, 12, 18).

The safety findings of this review also require balanced interpretation. Although adverse events were reported in 9.7% of patients on crude analysis, the pooled random-effects estimate was 14.6%, indicating that complications were not negligible. Reported adverse events included abdominal pain, fever, stent misdeployment, peritonitis and intra-abdominal infection. Serious events were uncommon, but their occurrence emphasizes that EUS-guided treatment of ALS should still be considered a technically demanding intervention that is best performed in experienced centers with access to radiologic, surgical and rescue endoscopic support (6, 9, 13, 15, 16). In addition, differences in adverse-event definitions and grading across the included studies limit direct comparability and argue for more standardized reporting in future series.

Another clinically relevant point is the position of EUS-guided bypass relative to alternative therapeutic approaches. Surgical revision has traditionally been regarded as definitive treatment, but is often undesirable in frail or palliative patients (1, 3). Percutaneous drainage may provide decompression, yet it is associated with external drainage devices and may negatively affect quality of life (6, 8, 29). Enteroscopy-assisted enteral stenting is another possible option, but may be technically challenging in altered anatomy and may not always provide durable relief (6, 7). Within this context, EUS-guided gastroenterostomy or enteroenterostomy appears particularly appealing because it provides internal decompression while preserving a minimally invasive approach (6, 9, 13).

This study has several limitations. First, the available evidence is based predominantly on retrospective case reports and case series, which are inherently subject to selection and publication bias. As successful clinical outcomes are disproportionately more likely to be published, this reporting bias potentially inflates the observed pooled technical and clinical success rates, meaning our findings may represent the upper bound of true efficacy. Second, outcome definitions were not completely uniform across studies, particularly with regard to clinical success and adverse events. Third, follow-up was highly heterogeneous and often limited, which inherently restricts our ability to draw firm conclusions regarding long-term outcomes. Specifically, critical parameters such as long-term stent patency, late symptom recurrence, exact reintervention rates, and the true durability of symptom relief were either completely omitted or inconsistently defined across the primary literature. Consequently, the lack of standardized reporting for these longitudinal metrics represents a significant limitation that curtails the overall clinical applicability of our findings regarding long-term maintenance. Fourth, as confirmed by our formal risk of bias and quality of evidence assessment, the overall body of evidence resides within a moderate-to-serious risk of bias, driven primarily by chronological selection bias and a lack of confounding control in the primary cohorts. This inherent methodological vulnerability underscores that the underlying data carries a low statistical certainty, further dictating a conservative interpretation of the pooled parameters.

Despite these limitations, the present review provides a clinically useful synthesis of the currently available evidence on EUS-guided treatment of ALS. The consistently high technical and clinical success rates across studies, together with an acceptable overall safety profile, support the use of EUS-guided transmural bypass as a valuable minimally invasive alternative to surgery or percutaneous drainage in appropriately selected patients (6, 9, 13). The absence of statistical heterogeneity (I^2^ = 0%) for primary outcomes likely reflects the uniformly high success rates across the published literature; however, it should be interpreted with strict caution. This statistical metric is a mathematical consequence of extreme event rates (ceiling effect) and should not mask the underlying clinical and procedural heterogeneity, including variations in stent types, diameters, patient populations, and diverse definitions of clinical success.

Future studies should focus on prospective multicenter data collection, uniform outcome definitions and longer follow-up. Comparative studies evaluating EUS-guided bypass against enteral stenting, percutaneous drainage or surgical revision would be particularly valuable in clarifying the optimal therapeutic algorithm for malignant and benign ALS. Further research should also better define the role of stent type, stent diameter and adjunctive measures such as coaxial double-pigtail stent placement.

In conclusion, EUS-guided transmural treatment holds potential as an alternative minimally invasive treatment option for ALS. Nonetheless, due to the fact that the current literature is restricted to small retrospective cohorts susceptible to publication bias, these favorable numbers must be interpreted with caution. Future prospective registries are essential to confirm these outcomes.

## Highlights

### What is already known

- Afferent loop syndrome (ALS) is an uncommon but clinically important complication after pancreaticobiliary or upper gastrointestinal reconstructive surgery.
- Surgical revision and percutaneous drainage may be invasive or poorly tolerated in frail patients, particularly in palliative settings.
- EUS-guided internal bypass has emerged as a minimally invasive alternative for selected patients with altered anatomy.

### What this study adds

- In this systematic review of 12 studies including 134 patients, EUS-guided gastroenterostomy or enteroenterostomy achieved high technical success (98.5%) and clinical success (94.8%), with an acceptable adverse-event rate (9.7%).
- The available evidence supports EUS-guided transmural bypass as an effective and reasonably safe treatment option for ALS, particularly in patients with malignant disease or increased operative risk.
