# Supplementary figures and images for: EUS-guided transmural treatment of afferent loop syndrome: a systematic review and meta-analysis
Source: Front Gastroenterol (Lausanne). 2026 Jul 14;5:1853386. doi: 10.3389/fgstr.2026.1853386 (PMC13407301; doi:10.3389/fgstr.2026.1853386)

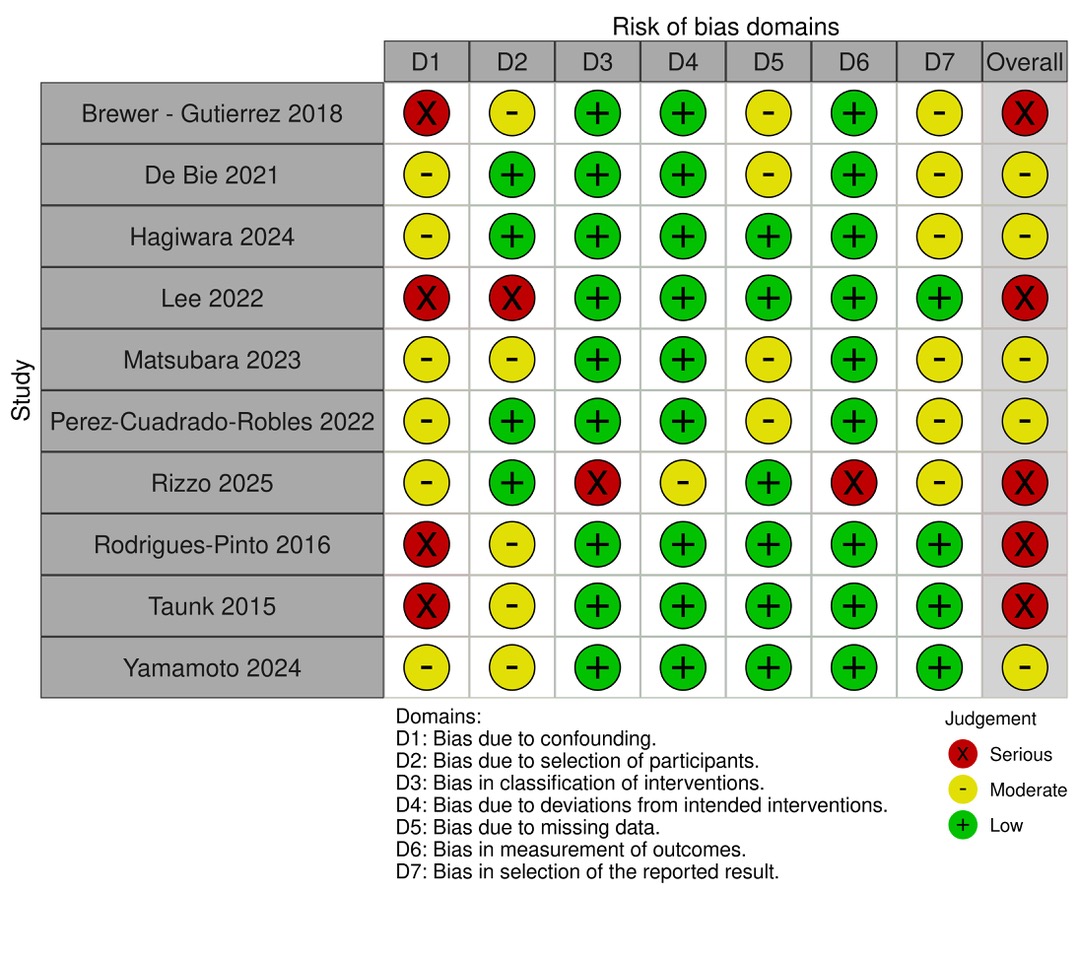

Supplement: Supplementary file 1 [file Image1.jpeg]
