# Supplementary material for: EUS-guided transmural treatment of afferent loop syndrome: a systematic review and meta-analysis
Source: Front Gastroenterol (Lausanne). 2026 Jul 14;5:1853386. doi: 10.3389/fgstr.2026.1853386 (PMC13407301; doi:10.3389/fgstr.2026.1853386)
Supplement: Supplementary file 2 [file Table1.docx]

**Supplementary Table S1. Full electronic search strategies**

*Databases searched: PubMed/MEDLINE, Embase, Cochrane Library. Date of last search: 15 May 2025.*

Search terms were combined using Boolean operators (AND/OR). [tiab] = title/abstract field tag (PubMed). :ti,ab = title/abstract (Embase). :ti,ab,kw = title, abstract, keyword (Cochrane). Searches were not restricted by language or publication date. Manual reference list screening was performed additionally for all included and relevant review articles.

**Database 1: PubMed/MEDLINE**

| **Search block** | **Boolean search string** |
| --- | --- |
| **Population (ALS):** | ("afferent loop syndrome"[tiab] OR "afferent limb syndrome"[tiab] OR "afferent loop obstruction"[tiab] OR "afferent limb obstruction"[tiab] OR "afferent loop"[tiab]) |
| **AND Intervention (EUS):** | ("endoscopic ultrasound"[tiab] OR "endoscopic ultrasonography"[tiab] OR "endosonography"[tiab] OR "EUS"[tiab] OR "EUS-guided"[tiab] OR "therapeutic EUS"[tiab]) |
| **AND Procedure:** | ("gastroenterostomy"[tiab] OR "gastrojejunostomy"[tiab] OR "enteroenterostomy"[tiab] OR "jejunojejunostomy"[tiab] OR "bypass anastomosis"[tiab] OR "transmural bypass"[tiab] OR "transmural drainage"[tiab] OR "lumen-apposing metal stent"[tiab] OR "lumen apposing metal stent"[tiab] OR "LAMS"[tiab] OR "EC-LAMS"[tiab] OR "self-expandable metal stent"[tiab] OR "SEMS"[tiab] OR "FCSEMS"[tiab]) |
| **Final combined query:** | (#1 AND #2 AND #3) — no date or language restrictions applied |

**Database 2: Embase (via Ovid)**

| **Search block** | **Boolean search string** |
| --- | --- |
| **Population (ALS):** | ('afferent loop syndrome':ti,ab OR 'afferent limb syndrome':ti,ab OR 'afferent loop obstruction':ti,ab OR 'afferent limb obstruction':ti,ab OR 'afferent loop':ti,ab) |
| **AND Intervention (EUS):** | ('endoscopic ultrasound':ti,ab OR 'endoscopic ultrasonography':ti,ab OR 'endosonography':ti,ab OR 'EUS':ti,ab OR 'EUS-guided':ti,ab OR 'therapeutic EUS':ti,ab) |
| **AND Procedure:** | ('gastroenterostomy':ti,ab OR 'gastrojejunostomy':ti,ab OR 'enteroenterostomy':ti,ab OR 'jejunojejunostomy':ti,ab OR 'bypass anastomosis':ti,ab OR 'transmural bypass':ti,ab OR 'transmural drainage':ti,ab OR 'lumen-apposing metal stent':ti,ab OR 'LAMS':ti,ab OR 'EC-LAMS':ti,ab OR 'self-expandable metal stent':ti,ab OR 'SEMS':ti,ab OR 'FCSEMS':ti,ab) |
| **Final combined query:** | (#1 AND #2 AND #3) — no date or language restrictions applied |

**Database 3: Cochrane Library (CENTRAL)**

| **Search block** | **Boolean search string** |
| --- | --- |
| **Population (ALS):** | ("afferent loop syndrome" OR "afferent limb syndrome" OR "afferent loop obstruction" OR "afferent limb obstruction"):ti,ab,kw |
| **AND Intervention (EUS):** | ("endoscopic ultrasound" OR "endosonography" OR "EUS" OR "EUS-guided"):ti,ab,kw |
| **AND Procedure:** | ("gastroenterostomy" OR "gastrojejunostomy" OR "enteroenterostomy" OR "jejunojejunostomy" OR "transmural bypass" OR "lumen-apposing metal stent" OR "LAMS" OR "self-expandable metal stent"):ti,ab,kw |
| **Final combined query:** | (#1 AND #2 AND #3) — no date or language restrictions applied |

**Abbreviations**

*ALS, afferent loop syndrome; EUS, endoscopic ultrasound; LAMS, lumen-apposing metal stent; EC-LAMS, electrocautery-enhanced lumen-apposing metal stent; SEMS, self-expandable metal stent; FCSEMS, fully covered self-expandable metal stent; PICO, population, intervention, comparator, outcome.*
