# Supplementary material for: EUS-guided transmural treatment of afferent loop syndrome: a systematic review and meta-analysis
Source: Front Gastroenterol (Lausanne). 2026 Jul 14;5:1853386. doi: 10.3389/fgstr.2026.1853386 (PMC13407301; doi:10.3389/fgstr.2026.1853386)
Supplement: Supplementary file 3 [file Table2.docx]

| ***Outcomes*** | ***No. of studies (design)*** | ***Risk of Bias*** | ***Inconsistency*** | ***Indirectness*** | ***Imprecision*** | ***Publication Bias*** | ***Certainty of Evidence*** |
| --- | --- | --- | --- | --- | --- | --- | --- |
| Technical Success | 12 studies(Observational) | Serious^a^ | Not Serious^b^ | Not Serious | Serious^c^ | Serious^d^ | ⊕◯◯◯ VERY LOW |
| Clinical Success | 12 studies(Observational) | Serious^a^ | Not Serious^b^ | Not Serious | Serious^c^ | Serious^d^ | ⊕◯◯◯ VERY LOW |
| Adverse Events | 12 studies(Observational) | Serious^a^ | Not Serious^b^ | Not Serious | Serious^c^ | Serious^d^ | ⊕◯◯◯ VERY LOW |

Supplementary Table S2. GRADE Evidence Profile for EUS-guided transmural treatment of afferent loop syndrome

^a^  **Downgraded by 1 level due to Risk of Bias:** All included primary data originate from retrospective case series and single case reports, which lack control groups and are highly susceptible to confounding and chronological selection bias (as mapped by the ROBINS-I tool).

^b^  **Not downgraded for Inconsistency:** Statistical heterogeneity was remarkably low (). While minor clinical heterogeneity exists regarding individual stent types or diameters, the directional magnitude of the effect (high success) was uniform across all cohorts.

^c^  **Downgraded by 1 level due to Imprecision:** The cumulative sample size across the entire published literature remains low (small number of total patients and events), which broadens confidence intervals under conservative statistical modeling.

^d^  **Downgraded by 1 level due to Publication Bias:** Strong evidence of reporting bias; single-arm retrospective surgical/endoscopic cohorts are inherently prone to the selective publication of successful outcomes ("ceiling effect"), while procedural failures remain disproportionately underreported.
